# Oxidative Stress in Preeclampsia and Placental Diseases

**DOI:** 10.3390/ijms19051496

**Published:** 2018-05-17

**Authors:** Rajaa Aouache, Louise Biquard, Daniel Vaiman, Francisco Miralles

**Affiliations:** 1 Institut National de la Santé Et de la Recherche Médicale, U1016, Institut Cochin, 75014 Paris, France; rajaa.aouache@inserm.fr (R.A.); louise.biquard@inserm.fr (L.B.); francisco.miralles@inserm.fr (F.M.); 2 Centre National de la Recherche Scientifique, UMR8104, 75014 Paris, France; 3 Département Développement, Génétique, Neurobiologie, Reproduction et Vieillissement, Université Paris Descartes, Sorbonne Paris Cité, 75014 Paris, France; 4 Département Hospitalo-Universitaire Risques et Grossesse, PRES Sorbonne, 75014 Paris, France

**Keywords:** preeclampsia, pregnancy, oxidative stress, placenta, vascular endothelium

## Abstract

Preeclampsia is a persistent hypertensive gestational disease characterized by high blood pressure and proteinuria, which presents from the second trimester of pregnancy. At the cellular level, preeclampsia has largely been associated with the release of free radicals by the placenta. Placenta-borne oxidative and nitrosative stresses are even sometimes considered as the major molecular determinants of the maternal disease. In this review, we present the recent literature evaluating free radical production in both normal and pathological placentas (including preeclampsia and other major pregnancy diseases), in humans and animal models. We then assess the putative effects of these free radicals on the placenta and maternal endothelium. This analysis was conducted with regard to recent papers and possible therapeutic avenues.

## 1. Introduction

### 1.1. Preeclampsia

Preeclampsia (PE) is a major disease of human pregnancy, marked by hypertension and proteinuria, appearing during the second or third trimester of gestation. Incidence differs depending on geographical region, time of year, nutrition, and race/ethnicity, but it affects roughly 3–8% of women worldwide [1,2,3]. Classically, PE is defined by de novo maternal hypertension (>140/90 mm Hg systolic/diastolic blood pressure) and proteinuria (>300 mg/24 h). In severe cases, the mother may develop comorbidities such as hepatic alterations (HELLP syndrome), edema, disseminated vascular coagulation (DIC), and eclampsia, particularly targeting the brain (cerebral edema). For the fetus, the main complications associated with PE include growth restriction leading to low birth weight (1/3 of cases), prematurity, and fetal death. From the onset of symptoms, the disease progressively worsens, potentially leading to both fetal and maternal demise. It remains one of the few fatal complications of pregnancy in industrialized countries today. To date, there is no cure for PE and in severe cases; it often requires premature labor induction, which carries with it the inherent risks for premature neonates.

Over the last decade, substantial progress has been made in understanding the pathophysiology of PE [4]. This originates from a defect of placental implantation into the maternal uterine wall. The impaired remodeling of the spiraled uterine arteries by the extravillous trophoblasts (EVT) leads to decreased placental perfusion. Consequently, intermittent arterial blood flow generates repeated ischemia/reperfusion episodes, thus creating a favorable environment for developing oxidative stress. Oxidative damage in the placenta leads to inflammation, apoptosis, and the release of cellular debris into maternal circulation, along with several anti-angiogenic factors, such as soluble fms-like tyrosine kinase-1 (sFlt1) and soluble Endoglin (sEng), cytokines, and oxidants. These placental-derived factors act on the maternal vascular endothelium, inducing oxidative stress and stimulating the production and secretion of pro-inflammatory cytokines, as well as vasoactive compounds. This results in a massive systemic endothelial dysfunction characterized by vascular inflammation and constriction [5]. Indeed, oxidative stress appears to be the central component of both placental and endothelial dysfunction, the causative etiology of PE.

### 1.2. Oxidative Stress

Oxidative stress (OS) is defined as “an imbalance between oxidants and antioxidants, leading to a disruption of redox signaling and control and/or molecular damage” [6]. OS involves reactive oxygen species (ROS), the most common being superoxide (O_2_^•−^), hydrogen peroxide (H_2_O_2_), and the hydroxyl radical (•HO). A parallel process is known as nitrosative stress (NS), which is defined as an imbalance in the ratio of nitrosants to antioxidants. NS principally involves the reactive nitrogen species (RNS) nitric oxide (•NO) and peroxynitrite (ONOO^−^). In aerobic organisms, the controlled production of ROS and RNS is a physiological phenomenon that plays a fundamental role in metabolism and cell signaling cascades. OS and NS occurs when there is an imbalance between the formation of oxidizing substances and the antioxidant molecules that promote their detoxification. Because of their highly reactive properties, ROS and RNS can cause structural and physiological damage to DNA, RNA, proteins, and lipids, including cell membrane-bound lipids.

**Figure 1:**
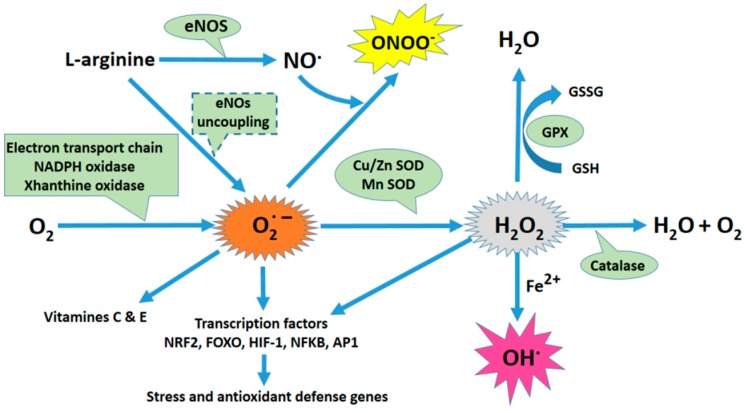
Oxidative stress plays a central role in the physiopathology of preeclampsia. Schematic of the principal sources of oxidative stress and its antioxidant mechanisms. The mitochondria, endoplasmic reticulum (ER), and nuclear membrane produce anions as a byproduct of the auto-oxidation of electron transport chain (ETC) components. ROS are also produced as a consequence of arachidonic acid metabolism by Cyclooxygenase 2 (COX-2), Lipoxygenases, Xanthine Oxydase (XO), and Cytochrome P450. NADPH oxidases (NOX) are another significant source of ROS. NOX generates superoxide (O_2_^•−^) by transferring electrons from NADPH inside the cell across its membrane and coupling them to O_2_. eNO synthase (eNOS) can generate O_2_^•−^ and H_2_O_2,_ specifically when the concentrations of its substrate, l-arginine, or its cofactor, tetrahydrobiopterin (BH4), are low. When intracellular ROS production increases (especially O_2_^•−^ ions), •NO may react with ROS to form peroxynitrite (ONOO^−^). Superoxide is rapidly dismutated to H_2_O_2_ by superoxide dismutase (SOD). H_2_O_2_ can be transformed into H_2_O and O_2_ by catalase and glutathione peroxidase (GPX). However, in the presence of Fe^2+^, and through Fenton’s reaction, H_2_O_2_ can generate the highly reactive radical hydroxyl (OH^•^).

Different cellular compartments or metabolic pathways can produce ROS and RNS (Figure 1). The mitochondria, endoplasmic reticulum (ER), and nuclear membrane produce O_2_^•−^ anions due to the auto-oxidation of components of the electron transport chain (ETC). ROS are also produced as a consequence of arachidonic acid metabolism by Cyclooxygenase 2 (COX-2), Lipoxygenases, Xanthine Oxydase (XO), and Cytochrome P450. Nicotin Amide Dinucléotide PHosphate oxidases (NOX) are yet another important source of ROS. The NOX generate O_2_^•−^ by transferring electrons from NADPH inside the cell across the membrane and reducing molecular oxygen. The NO synthase can generate O_2_^•−^ and H_2_O_2_, in particular when concentrations of its substrate, l-arginine, or its cofactor, tetrahydrobiopterin (BH4), are low. Additionally, when intracellular ROS production increases (especially O_2_^•−^), •NO may react with ROS to form peroxinitrite, ONOO^−^, a major cause of nitrosative stress [7].

Oxidative stress can also result from a lack of antioxidants [8]. There are two categories of antioxidants: enzymatic and non-enzymatic. The most relevant enzymatic antioxidants are superoxide dismutase (SOD), hemoxygenase (HO-1), catalase (CAT), glutathione peroxidase (GPx), and thioredoxin (TRX). SOD catalyzes the redox reaction of O_2_^•−^ into O_2_ and H_2_O_2_. H_2_O_2_ is quickly neutralized by CAT, which then breaks it down into H_2_O and O_2_. GPx, an enzyme dependent on the micronutrient selenium (Se), plays a critical role in the reduction of hydrogen and lipid peroxides. This enzyme uses glutathione (GSH) as a cofactor to reduce H_2_O_2_, resulting in the formation of oxidized glutathione (GSSG). TRX is an oxidoreductase enzyme that facilitates the reduction of other proteins through the formation of disulfide bridges between cysteine residues. GSH, vitamins C and E, Nicotinamide adenine dinucleotide (NADH), and Nicotinamide adenine dinucleotide phosphate (NADPH) are all non-enzymatic antioxidants. GSH neutralizes ROS (especially H_2_O_2_) and plays a role in maintaining vitamins C and E in their reduced form. Among other functions, NADPH is involved in the protection against ROS by allowing the regeneration of GSH. Since OS can occur in different cellular compartments and through different mechanisms, the capacity of antioxidants to block either the ROS or RNS strongly depends on their local production (or import) and the nature of the oxidative stress.

Among its multiple effects on cell physiology, OS leads to the regulation of transcription factors such as AP-1, NRF2, FoxO, CREB, HSF1, HIF-1α, TP53, NF-κB, Notch, SP1, and CREB-1. Of these, only NRF2 and FoxO control the expression of several genes that encode enzymes required for the detoxification of oxidizing molecules.

## 2. Major Enzymatic and Cellular Systems Involved in the Generation of Free Radicals

Radical species (ROS and RNS) play a central role in cell physiology as second messengers in many signaling pathways [9]. In addition, radical species play a major role in the initiation of placental and endothelial dysfunction: two major features of PE [9,10]. The principal sources of radical species in this case are the NO synthases (NOS), the NADPH oxidases (NOX), the mitochondrial electron transport chain (ETC), and xanthine oxidase (XO).

### 2.1. Nitric Oxide (NO) Synthases

•NO is synthesized from l-arginine under the action of Nitric Oxide Synthetases (NOS), which catalyze l-arginine oxidation into •NO and l-citrulline [11,12]. There are at least 3 NOS proteins encoded by distinct genes. Neuronal nitric oxide synthase (nNOS), which is constitutively expressed in specific neurons of the brain; inducible nitric oxide synthase (iNOS), whose expression is typically induced during inflammatory disease states; and endothelial NOS (eNOS), also known as constitutive NOS. NOS is an enzyme formed by a homodimeric complex that includes a domain with reductase activity and one with oxidase activity. The production of •NO results from the succession of two reactions: the first uses molecular O_2_ to hydroxylate l-arginine to N^ω^-hydroxyl-l-arginine. The second involves the oxidation of NG-hydroxyl-l-arginine, thus forming •NO and l-citrulline. Both reactions require the presence of the cofactor tetrahydrobiopterine (BH4). When intracellular O_2_^•−^ production increases, •NO may also react with it, forming ONOO^−^. This is a powerful oxidant that can modify proteins and lipids by nitration. Additionally, peroxynitrite oxidizes BH4, resulting in the inactive molecule BH2 [13]. In the absence of BH4, eNOS shifts from a homodimeric to a monomeric form, thus becoming uncoupled [14]. In this free conformation, it does not synthesize •NO, but instead generates O_2_^•−^. In ECs, this uncoupling of eNOS has two important consequences: a loss of vasodilation due to the loss of •NO, and an increase in oxidative stress by the formation of O_2_^•−^. Consequently, any dysfunction in the regulation of eNOS activity can have profound metabolic consequences. Control of eNOS activity involves a complex regulatory mechanism that includes an inhibitory interaction with Caveolin (CAV1), post-translational modifications (myristoylation, palmitoylation, and phosphorylation), sub-cellular compartmentalization, and stimulatory responses to rises in intracellular calcium levels, reviewed by [15].

### 2.2. Role of the NADPH Oxidase (NOX) as a Source of ROS

NOX are a family of transmembrane proteins that transfer electrons from NADPH across the membrane to O_2_, thus forming O_2_^•−^. Seven members comprise the NOX family (reviewed by [9,10,16]). The paradigmatic NOX family member is NOX2, which requires the cytosolic proteins p47phox, p67phox, and p40phox for activity, as well as the membrane-associated p22phox, and gp91phox (now named NOX2). In contrast, NOX4 only requires the membrane protein p22phox, and its activity seems to be constitutive and regulated at the expression level. NOX1, 2, 4, and 5 are all expressed in vascular tissue but with variable expression levels between cell types. NOX4 is the most abundant in blood vessels, particularly in ECs.

Multiple studies have consistently demonstrated that hypertension is associated with an increase in OS, partly via NOX deregulation [9]. In many models of hypertension, the link between blood pressure regulation and NOX activity has been well established. For example, angiotensin II (AngII) infusion produces hypertension in animal models. Moreover, this process is dependent on increased NOX-derived O_2_^•−^ production throughout the vascular wall, which leads to cellular dysfunction in ECs. Most of these studies, however, were focused on the NOX isoforms that generate O_2_^•−^, which can impair •NO-dependent vasodilation [9]. On the contrary, it appears that overexpression of NOX4 in the endothelium induces vasodilation through H_2_O_2_ production and increases eNOS protein expression [17].

### 2.3. Mitochondrial Reactive Oxygen Species (ROS) Production

Mitochondria are a major source of ROS production [18,19]. Most mitochondrial ROS is produced by the complexes of the electron-transport chain (ETC). During oxidative phosphorylation, electrons are transferred from electron donors (NADH), produced by the Krebs cycle, to electron acceptors such as O_2_, through redox reactions. These reactions are carried out in the ETC, which is a series of five protein complexes located in the folded inner membrane of the mitochondria. During this process, a small percentage of electrons can leak out and react with O_2_ to produce O_2_^•−^. Superoxide that does not escape the mitochondria is reduced to H_2_O_2_ by the manganese superoxide dismutase (MnSOD) and copper-zinc superoxide dismutase (CuZnSOD) in the matrix and inter-membrane space, respectively. H_2_O_2_ may then either leave the mitochondria, react with mitochondrial proteins, or be reduced to H_2_O by local peroxidase enzymes.

### 2.4. Xanthine Oxidase and ROS

Xanthine oxidase is a form of the enzyme Xanthine oxidoreductase (XOR). This enzyme exists as two forms: xanthine dehydrogenase (XDH) and xanthine oxidase (XO). These enzymes catalyze the oxidation of hypoxanthine to xanthine and then can further catalyze the oxidation of xanthine to uric acid. These reactions produce two molecules of H_2_O_2_ and two molecules of O_2_. XOR is expressed primarily in the liver and small intestine, but is also present in ECs and in the circulation. Increased activity of XO has been found in cases of airway inflammatory disorders, ischemia-reperfusion injury, atherosclerosis, diabetes, and autoimmune disorders [20].

## 3. Cellular Sources of Oxidative Stress in the Human Placenta under Normal and Pathological Conditions

### 3.1. Cell Types of the Placenta and the Origins of Oxidative Stress

**Figure 2:**
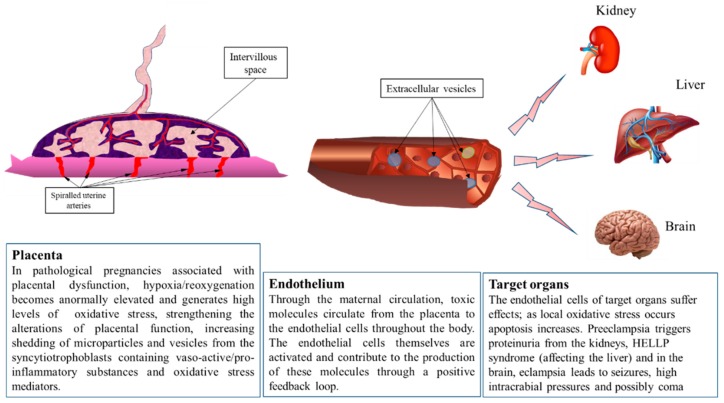
The placenta is the interface between mother and fetus. The placenta plays an indispensable and multifunctional role as the interface between the two adjoined organisms, regulating immunological dialogue and tolerance, nutrient and gas exchange, and producing hormones essential for pregnancy. The first trimester of gestation in humans is characterized by general hypoxia in the intervillous space, as many maternal spiral arteries are plugged with extravillous trophoblast cells, preventing maternal red blood cells from passing into this space. After 12–14 weeks of normal gestation, these placental extravillous trophoblasts colonize the maternal spiraled arteries as far as the proximal third of the myometrium. This innervation leads to the arteries to relax and lose contractile properties, which increases blood flow and raises oxygen partial pressure, thus reversing the previously hypoxic environment. Therefore, oxidative stress normally occurs in the healthy placenta and may in fact be important for its organogenesis. However, in cases of incomplete placentation, this stress occurs at an excessive level and leads to an elevated release of placental debris and vesicles into the maternal circulation. These extracellular vesicles carry active molecules (proteins of microRNA) generated by stressed placental cells. Once in the blood, they meet maternal endothelial cells and potentially transfer their contents, leading to transcriptome alterations and inflammation. Eventually, the endothelium of maternal organs is affected. In the case of preeclampsia, this most heavily occurs in the kidney, liver, and brain.

The human placenta is composed of numerous cell types, as recently shown by single-cell sequencing performed by Tsang et al. [21] that showed twelve major cell clusters identified by their expression profile. Among them, the most abundant are stromal cells, endothelial cells, villous cytrophoblasts (CTV), and extravillous trophoblasts (EVT). In addition, the syncytiotrophoblast (SCT), resulting from the fusion of villous trophoblasts, was clearly separated from other cells of the trophoblast lineage. Syncytiotrophoblasts are characterized by their synthesis of an mRNA that encodes the Chorionic Gonadotrophin β polypeptide, which, together with Chorionic Gonadotrophin α polypeptide, constitutes human Chorionic Gonadotrophin (hCG) hormone. hCG is synthesized by the placenta as early as implantation and syncytialization, approximately 8 days post-fertilization in humans. Interestingly, clusters of immune cells were also identified (Macrophages, Dendritic cells, T cells), which may be responsible for generating oxidative stress. Oxidative stress can be induced by low oxygen partial pressure, hyperoxia, or alternations of hypoxia and reoxygenation, as seen in various highly vascularized tissues such as the brain or eye [22,23]. In the normal placenta, hypoxia is a physiological condition detectable during the first trimester (in humans), as measured by in vivo methods [24]. This normally occurring phenomenon blurs the distinction between regular physiology and possible detrimental effects of hypoxia at specific stages of placental development. Aside from trophoblast cells, oxidative stress could originate in endothelial cells (ECs) present in the placental tissue, stromal cells of the villi, or immune cells (Hofbauer cells). Generally, trophoblasts are modified due to oxidative stress by way of gene expression. This is achieved by the placenta, which generates extracellular vesicles that are released into maternal circulation and will influence gene expression in both maternal endothelial and immune cells (Figure 2). These extracellular vesicles are exosomes, measuring 30–100 nm, and microvesicles at 100–1000 nm. Larger cell debris is also released by the placenta, which includes nuclear aggregates and apoptotic bodies (20–500 µm and 1–4 µm, respectively). These events also occur and are most likely enhanced in the context of PE, as recently shown by Verma et al. [25].

### 3.2. Oxidative Stress from the Trophoblasts

To elucidate the origin of oxidative stress, many studies have been carried out on trophoblast cell models, specifically HTR8/SVneo cells that had been exposed to hypoxia/reoxygenation (H/R). The HTR-8/SVneo cell line was developed from first trimester extra-villous trophoblast infected with SV40 Large T antigen. Despite recent evidence that it may be a heterogeneous cell line, also including pure trophoblasts (marked by CK-7) and mesenchymal cells (marked by vimentin) [26], it has been extensively used as a sufficient model of extravillous trophoblasts. Recent papers have shown, for instance, that oxidative stress applied in the context of this model induces down-regulation of *N*-acetylglucosaminyltransferase III [27], Special AT-rich sequence Binding protein 1 (SATB1, localized in placental trophoblast cells [28]), and abnormal regulation of Parkinson Associated deglycase 7 (PARK7) [29]. SATB1 has been shown to inhibit the Wnt-β-Catenin pathway, and thus the migration and invasion of the trophoblast. H/R treatment in HTR-8/SVneo cells also induces alterations of the Phospho Inositol 3 Kinase/Protein Kinase B (AKT) pathway. Together with serum deprivation, it triggers an increase in necrotic cell death, which was able to be rescued by relaxin, an insulin superfamily hormone expressed by the maternal reproductive system. It has also been shown that trichloroethylene (TCE) or flame retardants such as Polybrominated diphenyl ethers (PBDE, such as BDE-47) do indeed modify the response to oxidative stress in this model [30,31]. The TCE metabolite DCVC (S-(1,2-dichlorovinyl)-l-cysteine) induces inflammation by releasing IL-6 following the generation of ROS, as a result of its disrupted mitochondrial function.

In these trophoblast cell lines, antioxidant molecules have proven to be able to reverse oxidative stress cascades. This is the case for Resveratrol [32], which normalized the activity of SOD in the HTR8-SVneo H/R stressed cells, as well as the concentration of Malondialdehyde (a marker of oxidative stress targeting unsaturated fatty acids), and decreased apoptosis. Similarly, Sildenafil citrate (Viagra) applied to H/R HTR-8/SVneo cells can promote cell survival, if in the presence of NO and cGMP [33]. Oxidative stress-induced apoptosis can also be attenuated by HBEGF treatment in first trimester placental explants, as well as on the HTR-8/SVneo cells [34]. In these cells, exposure to oxygen peroxide induced down-regulation of HLA-G expression, a trophoblast immunomodulatory molecule that facilitates implantation and placental development [21]. It normally leads to proliferation inhibition, apoptosis, and decreased cell invasion [35]. Therefore, through exposure to OS, the EVTs of the placenta may trigger an excessive immune response via the decidua cells. This can lead to a decreased endometrium invasion and faulty uterine spiraled artery assembly, the latter being a hallmark feature of preeclampsia. Consistently, Preimplantation Factor (PIF*), a 15 amino-acid linear peptide secreted early by viable embryos, was discovered as an enhancer of implantation and has recently been shown to protect against oxidative stress, thereby enhancing HLA-G expression and fostering better implantation [36].

### 3.3. Oxidative Stress Origin and Regulation in Hofbauer Cells

There is but a handful of papers that address non-trophoblast placental cells and oxidative stress. As early as 1998, the team of Graham Burton in Cambridge revealed that in the placental stroma, Hofbauer cells (placental macrophages) express catalase at early stages of human pregnancy (6–17 weeks post-fertilization). While the enzyme was hardly detected in the SCT [37], catalase is indeed a major enzyme for the detoxification of hydrogen peroxide (H_2_O_2_). The authors interpreted the low levels as an indication of an equally low level of oxidative stress in the young human placenta. These results may also suggest a potential role of Hofbauer cells in the detoxification of oxidative stress. In 2013, Sisino et al. studied the effect of hyperglycemia on Hofbauer cells in human placentas and rat models [38]. They show that the cells move from an M2 to an M1 phenotype (pro-inflammatory, inducing CD68, CCR7, and IL1β, and decreased CD163, CD209-aka DC-SIGN1, and IL10) while accompanied by an activation of oxidative stress pathways and a HIF1α-mediated high-glucose response. The authors isolated Hofbauer cells from human placentas and treated them with 25 mM of glucose, inducing the transcription of HIF1α and NOS2 and leading to a significant increase of NO in the culture medium. This study, which initially aims to understand the function of placental cells in gestational diabetes, suggests that Hofbauer cells may also be a source of NO in the context of other pathologies. Thus, they may also contribute to the generation of Nitrosative Stress through the creation of peroxynitrite in the presence of oxidative stress, even if induced by other cell types. Conversely, a different study suggests a putative link between Hofbauer cells and down-regulation of oxidative stress. Holwerda et al. were interested in the dynamics of hydrogen sulfide (H_2_S) during the pathogenesis of preeclampsia [39,40]. Simlarly to NO, this molecule has pro-angiogenic and anti-oxidative properties [41,42], and is regulated by two major enzymes: cystathionine-γ-lyase (CSE) and cystathionine-β-synthase (CBS). H_2_S induces vasodilation via the inhibition of calcium-dependent K+ channels in the vessel smooth muscle cells, and induces the pro-angiogenic growth factor, VEGF. Its action on *VEGF* expression may be mediated by the down-regulation of miR-20a, miR-20b, miR-200c, and MiR-133b [43,44]. Both CSE and CBS are expressed in the endothelial cells of the placenta and decidua, while CBS appears to also be expressed in Hofbauer cells. In a previous study, we showed that maternal preeclamptic plasma is able to decrease the expression of CBS in endothelial cells [45], suggesting that Hofbauer cells could induce an antioxidant mechanism that modulates oxidative stress and reduces its effects. Therefore, at the placental level, it appears that the system governing hydrogen sulfide down-regulation through CSE regulation could be an important therapeutic possibility in the context of preeclampsia [46].

### 3.4. Consequences of Oxidative Stress on the Physiology of Placental Cells

#### 3.4.1. Cell Models

Extensive cellular and animal models have been used to evaluate the effects of oxidative stress on the placenta. Several aspects of cell physiology have been analyzed including proliferation, apoptosis, migration, syncytialisation, and nutrient transport. For instance, on TCL1 cells, a model of EVT, exposure to X/XO, and 0.1 mM of H_2_O_2_ induces a significant decrease in proliferation and inhibits the invasion and capability to form tube-like structures in these cells. Terminal dideoxynucleotidyl transferase dUTP Nick End Labeling (TUNEL) assays indicate that oxidative stress increases apoptosis in TCL1 cells. Additionally, exposure to H_2_O_2_ also has pro-apoptotic and anti-proliferative effects on isolated human cytotrophoblasts [47].

Choriocarcinoma BeWo cells are a recognized cell model of villous trophoblasts cells (CTV). Upon exposure to forskolin, these cells fuse, form a syncytium, then produce and secrete hCG. A comparative study of BeWo cells under hypoxic (2% O_2_) or normoxic (20% O_2_) conditions indicates that hypoxia decreases cell proliferation, as well as its fusion into SCT and HCG secretion [48]. The impaired rate of fusion could be explained by a decrease in expression of both syncytin and amino acid transport system B(0), which are crucial for implantation. The same experiments have been performed on choriocarcinoma cells, JEG-3, which have been classically used as an extravillous trophoblast (EVT) cell model. JEG-3 cells were exposed to H_2_O_2_ (10 to 500 µM) then evaluated for changes in proliferation, apoptosis, and hCG secretion. It was found that the cells treated with H_2_O_2_ displayed a dose-dependent decrease of hCG secretion. Exposure to strong concentrations of H_2_O_2_ also changed the phenotypes of JEG-3 cells [49]. They became round and retractile, detached from cultures dishes, and displayed chromatin condensation and nuclear fragmentation. JEG-3 cells exposed to oxidative stress resulted in increased TUNNEL labelling, which is indicative of a higher rate of apoptosis. The apoptotic process seems to be mediated by the activation of Extra cellular Regulated Kinase (ERK)1/2, p38 Mitogen Activated Protein Kinase(MAPK), and c-Jun N-terminal kinase (JNK) protein [50]. This phenomenon has also been observed in explants of villous trophoblasts cultured in hypoxic conditions (2% O_2_). Under these conditions, the expression of *BCL-2* mRNA (an inhibitor of apoptosis) is decreased, whereas the expression of apoptotic activators (Bax and Bak) is increased [51].

A recent study has explored the role of oxidative stress and C/EBPβ in the context of PE and its involvement in regulation of the invasive capacities of trophoblastic cells [52]. Elevated C/EBPβ and low β-catenin expression has been found in preeclamptic placentas compared to non-pathologic samples, and a similar situation has also been observed in villous explants cultured under hypoxia/reoxygenation. The EVT cell line HTR8/SVneo, when exposed to the same conditions, also showed increased C/EBPβ. Moreover, knockdown of C/EBPβ significantly increased β-catenin expression and promoted the invasive capacities of HTR8/SVneo cells. It also enhanced the outgrowth and migration of villous explants and inhibited the excessive generation of intracellular ROS. These findings could be attributed to increased activity of the metalloproteinases MMP-2/9 and decreased expression of TIMP-1/2. The authors hypothesize that the oxidative, stress-induced overexpression of C/EBPβ might influence the activity of MMPs by regulating the Wnt/β-catenin signaling pathway, thereby restraining the invasive capacities of trophoblast cells and contributing to the development of PE.

Oxidative stress has also been involved in alterations of the transport mechanisms in trophoblastic cells. For instance, ROS, including H_2_O_2_, have been shown to have an effect on the Ca²^+^ placental transporter polycystin-2 (PC2). Specifically, patch clamp experiments have demonstrated that ROS and peroxidized lipids inhibit PC2 activity in trophoblast membranes. Since PC2 activity prevents Ca²^+^ overload in placental cells, the modification of Ca²^+^ transport has the potential to severely impact placental function [53].

Elevated plasma levels of uric acid are found in preeclamptic women and have been associated with restricted fetal growth in utero. Although normally considered a marker of impaired renal function, it also has deleterious effects on cytotrophoblasts physiology [54]. A key feature of uric acid is its ability to act either as an antioxidant or pro-oxidant depending on a variety of factors, thus rendering its biological effects extremely pleiotropic. A study using primary placental villous explants has shown that the amino acid uptake, specific to the System A transporter, is reduced in a concentration-dependent fashion with increasing levels of uric acid. However, uric acid-induced reduction in System A activity can be partially reversed by NOX inhibition and completely eliminated by antioxidant treatment. Therefore, uric acid inhibits the placental System A amino acid transport most likely through stimulation of intracellular redox signaling cascades. Hyperuremia has a detrimental effect on amino acid transport in the placenta and can thus contribute to the pathophysiology of PE and restricted fetal growth.

#### 3.4.2. Animal Models

Various in vivo models of PE point also to oxidative stress as an inductor of apoptosis in the placenta. Pregnant mice treated with sFlt1, for example, show histological modifications in the placenta and uterus. These include swelling and hypercalcification of the villous vessels and increased thickness of the uterus glands [55]. These phenotypic modifications could be due to an increase in oxidative stress induced by sFlt1. Indeed, mouse blood levels of Malondialdehyde (MDA), a maker of oxidative stress, are significantly increased, whereas SOD levels are decreased. Electron microscopy of control and treated mouse placentas shows swollen mitochondria in the trophoblasts of mice treated with sFlt1, and TUNEL assays revealed an increased number of apoptotic cells. It was also observed that the expression of apoptotic Bax and cleaved Caspase 3 proteins was up-regulated, while BCL-2 was down-regulated. The authors proposed the hypothesis that a cascade of negative effects is induced by sFlt1, as this factor potentially leads to increased oxidative stress on trophoblast cells, which then promotes morphological and functional damages and, finally, apoptosis.

When severe PE was induced by STorkhead bOX protein 1 (*STOX1*) overexpression in the mouse placentas [56], a dramatic shift in the balance between OS and NS was observed, as shown by the labeling of carbonylated and nitrosylated proteins [57]. This was accompanied by a very clear alteration of gene expression in the mitochondria and of the mitochondrial mass itself. This translated into a perturbation of ATP production, and a possible exhaustion of free NO, thereby leading to endothelial dysfunction.

Beausejour et al. created a rat model of PE, whereby they injected a solution of NaCl (0.9% or 1.8%) during the last week of gestation [58]. In this condition, the placentas of these animals show an increase in 8-isoPGF2-α, TXB2, TNF-α, and eNOS expression, and a decrease in total GSH levels. These findings are indeed indicative of elevated oxidative stress in preeclamptic mice. In addition, this model also demonstrated an increase in the apoptotic index (Bax/Bcl-2) ratio and number of apoptotic cells.

### 3.5. Consequences of Oxidative Stress on Protein Post-Translational Modifications, Lipid Alterations, and DNA Damage in the Placenta

A recent paper examined the links between SUMOylation in the placenta and oxidative stress [59]. SUMOylation is defined as the covalent linking of SUMO, a ~100 amino-acid protein generally bound to a lysine inside a specific tetrapeptide, through the action of conjugating enzymes, such as UBC9 [60,61]. Baczyk et al. showed that amongst the 4 SUMO isoforms, subcellular localization of these post translational markers evolve during pregnancy, under the influence of partial oxygen pressure (0.5% O_2_) and oxygen peroxide. This study is the first to link a major molecule of oxidative stress with SUMOylation. This rare work directly studied the effect of oxidative stress in different cell types on placental explants, via analysis of different SUMO proteins. Specifically, SUMO1 and 4 were restricted to the CTV during the first trimester, then re-localized to the SCT at term, while SUMO2 and 3 were evenly distributed in the trophoblast but increased under oxidative stress. This condition caused SUMO1 and 4 to increase in the SCT, while SUMO2/3 increased in the nuclei. The authors state that these PTM could potentially impact the cytoskeleton, and thereby promote shedding of the SCT, a hallmark feature of preeclampsia [59].

One of the most frequent post-translational modifications observed in the placenta is associated with nitration, i.e., the transfer of a nitrogen species on the tyrosine residues of proteins. This is not necessarily specific to placental proteins, but rather to a very general mechanism known to have huge impacts in the context of human disease, for example, in cardiovascular dysfunction [62]. This modification that leads to nitrotyrosine production is directly associated with NS, which plays a major role in the context of PE [63,64]. Nitration may in fact target specific proteins and interfere with their activity, as is the case with p38 MAP Kinase [65]. Peroxynitrites, on the other hand, can have inverse effects on specific proteins that are important for placental physiology, such as MMP-2 and MMP-9. This phenomenon is observed, for instance, in the placentas of patients with Type II Diabetes [66]. There is experimental evidence that shows peroxynitrites can also alter the endothelial expression of vasoregulatory factors and thus induce placental vascular dysfunction [67].

In preeclamptic placentas, lipid peroxidation and protein carbonyls are significantly increased compared to non-pathological placentas [68,69]. This could be due to an increase of oxidative stress [68,70,71], or a decrease of antioxidant enzyme activities such as SOD, GPx and TRX [68]. Carbonyl levels are even more clinically significant in preeclamptic patients who also have HELLP syndrome [69]. Oxidative stress can also induce the accumulation of advanced oxidation protein products (AOPPs), which could have an effect on trophoblast cells and lead to delevopment of PE. In fact, HTR8/SVNeo cells treated with different concentrations of AOPPs display an increase in Sflt1 mRNA and protein expression in a dose dependent manner. These effects are able to be inhibited by a combination of apocynin and NOX inhibitors. Therefore, accumulation of AOPPs might contribute to the pathogenesis of preeclampsia by promoting sFlt-1 production in trophoblasts [72].

Additionally, oxidative stress can also induce nucleic acid damage. Immunohistochemical labelling with γH2AX (a marker of DNA double-strand breaks) showed an increased number of γH2AX-positive cells in preeclamptic placentas, especially in decidual cells, as compared to non-pathological placentas [73]. Indeed, preeclamptic placentas with high levels of the oxidative stress marker MDA also display an increase in DNA fragmentation that can lead to trophoblast apoptosis [70]. Primary cultures of trophoblast and decidual cells treated with 100 µM H_2_O_2_ show significant DNA damage, including double strand breaks. This damage could very likely affect the biological function or transcriptional regulation of placental genes involved in implantation, placentation, and cell fate decision [73]. Other studies have shown that cellular concentrations of 8-Hydroxy-2′-deoxyguanosine (8-OH-dG), a marker of oxidative DNA damage, are significantly higher in placental DNA from preeclamptic patients with IUGR, as compared to normal placentas [74,75].

### 3.6. Oxidative Stress in Placental Pathologies Other than Preeclampsia

**Figure 3:**
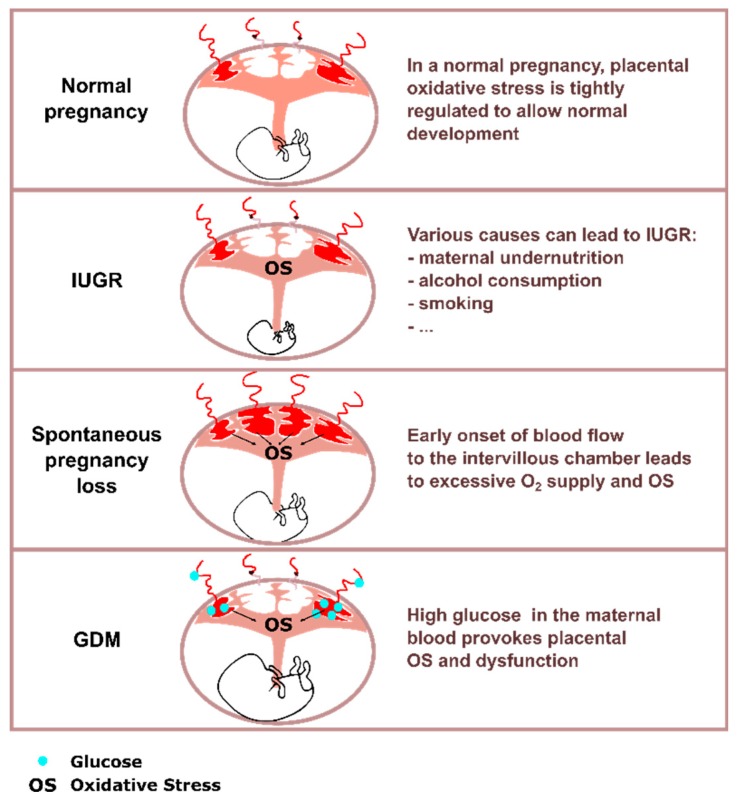
Schematic representation of first trimester placentas in IUGR, GDM, and spontaneous pregnancy loss. Oxidative stress (OS) is a key characteristic in various placental pathologies. Although the causes of IUGR can be numerous, placental oxidative stress is recurrently found. In the case of spontaneous pregnancy loss, initial onset of the blood flow in the intervillous chamber happens earlier and is less organized than in normal pregnancies, which leads to an increase in oxidative stress in the placenta. In gestational diabetes, variations in maternal glycaemia participate in placental oxidative stress induction. In all the pathologies mentioned, placental oxidative stress leads to various dysfunctions. Key: glucose (blue dots), oxidative stress (OS), Intra-Uterine Growth Restriction (IUGR), and Gestational Diabetes Mellitus (GDM).

In addition to PE, oxidative stress is involved in a number of placental pathologies including spontaneous pregnancy loss, intra-uterine growth restriction (IUGR), and gestational diabetes (Figure 3).

#### 3.6.1. Spontaneous Pregnancy Loss

Spontaneous pregnancy loss is a common pathology; about 15% of all clinically recognized pregnancies result in pregnancy failure [76]. The onset of blood flow in the intervillous space occurs between the 10th and the 12th week of pregnancy, accompanied by an increase in the activity of placental antioxidant enzymes [77]. When comparing cases of miscarriage vs. healthy pregnancies at 8–9 weeks by Doppler ultrasonography, Jauniaux et al. found that maternal-placental blood flow was already established in the abnormal pregnancies, therefore exposing the placenta to an excess of oxygen at stages when physiological hypoxia is primordial [78]. Differences in markers of oxidative stress in placentas have been consistently reported, as well as increased apoptosis and decreased proliferation of trophoblast cells [79,80,81]. Transcriptomic analysis showed a decreased expression of genes involved in mitochondrial function in the villi of patients suffering from pregnancy loss as compared to normal pregnancies [82]. However, unsupervised clustering was unable to efficiently segregate the samples of unexplained miscarriage and control pregnancies, underlining the etiological complexity of unexplained miscarriage.

Indeed, the molecular effects of placental oxidative stress in early pregnancy loss remain elusive. It is known, however, that in primary cultures of decidual cells from patients with early pregnancy losses, ER stress is higher and the Unfolded Protein Response (UPR) is less efficient [83]. The same group also mechanistically demonstrated that H_2_O_2_ treatment provokes ER stress in normal decidual cells and can also inhibit the UPR at high concentrations, which is a prerequisite for ER stress alleviation [84]. This suggests that the increase of oxidative stress in placentas of pregnancy loss could mediate ER stress and inhibit the UPR.

The implication of placental oxidative stress in certain cases of embryonic resorption has been demonstrated in rodent models in which the strain is especially prone to this phenomenon (CBA/J × DBA/2), in conjunction with increased lipid peroxidation and decreased enzymatic activity of key antioxidants [85]. In a systemic oxidative stress model expressing a defective ECT complex II, the number of embryonic resorption at the time of first delivery was significantly increased with a visible inflammation of the placenta [86]. However, the development of a pure model of placental oxidative stress would be most relevant for assessing whether this stress is sufficient to induce embryonic resorption.

#### 3.6.2. Intra-Uterine Growth Restriction (IUGR)

IUGR is a common pregnancy pathology diagnosed when the fetus weight is under the tenth percentile compared to standard weights at a given gestational age [87]. IUGR can have various causes such as maternal malnutrition, excessive alcohol consumption, smoking, and also glucocorticoid treatment. It is often seen with comorbidities such as PE and gestational diabetes mellitus (GDM). In humans, IUGR is associated with elevated oxidative stress in the placenta [81,88]. Transcriptomic analysis showed a significantly decreased expression of genes involved in mitochondrial function and oxidative phosphorylation (NADH dehydrogenase, cytochrome C oxidase Va, ubiquinol-cytochrome C reductase [89]).

In rodent models, different etiologies of IUGR all lead to oxidative stress in the placenta, confirming that oxidative stress is extremely relevant in the pathophysiology of this disease. In mice, when IUGR is induced by ischemia-reperfusion cycles of the placenta (temporary occlusion of uterine arteries), 8-OHdG DNA damage provoked by oxidative stress is present [90]. In a dexamethasone-induced model of IUGR, no difference was detected in classical oxidative stress pathways, but a strong increase of antioxidant enzymes activity was observed, implying that the placenta adapted to its pro-oxidant conditions [91]. More indirect evidence of oxidative stress involvement in IUGR is the cellular prion protein knock-out and overexpression mouse model, which both develop severe IUGR [92]. The prion protein is a Cu^2+^ chelator [93] and could thereby contribute to the activity the Cu/Zn SOD, thus affecting the metabolism of H_2_O_2_ and superoxide ions (O_2_^•−^).

Methylome data obtained on human IUGR full-term placentas clustered by GO analysis showed markers associated with oxidative stress, autophagy, and hormonal responses [94]. Autophagy is an important catabolic process that degrades damaged cellular organelles and proteins. It is important for cellular homeostasis and survival in stressful conditions, but when it is over-activated, it can result in cell death [95]. Moreover, oxidative stress has been found to regulate autophagy [96]. In term placentas of women with IUGR, the number of autophagy vacuoles was found to be increased and localized at the trophoblast layer of the placenta [97]. Additionally, when a BeWo cell line was cultured in 6% oxygen and 5% serum—conditions susceptible to lead to oxidative stress—the presence of autophagosomes was detected in the treated condition, but not the control [97]. These results are consistent with the hypothesis that increased deleterious autophagy is due to oxidative stress in placentas of women with IUGR.

#### 3.6.3. Gestational Diabetes Mellitus

Gestational diabetes mellitus (GDM) is a pathology defined by any degree of hyperglycemia that is recognized for the first time during pregnancy. Analogous to type-2 diabetes mellitus, systemic oxidative stress has been associated with GDM.

Despite some discrepancies in humans, likely attributable to heterogeneity in patient recruitment and GDM management, GDM has been widely associated with disturbed levels of OS and NS markers in the placenta. In the Wistar rat, placentas of diabetic females at day 17 of gestation showed onset of diabetes by streptozotocin injection, which led to an increase of TBARS (Thiobarbituric Acid Reactive Substances), a decrease in the GSH/GSSG ratio, indicative of antioxidant defense, and DNA oxidation adduct 8-oxo-dG, which is the classical mark of oxidative stress on DNA [98].

Several in vitro studies suggest that hyperglycemia is a direct cause of oxidative stress variations in placentas of GDM patients. In humans, the placenta is directly in contact with the maternal blood supply during the second and third trimester of pregnancy. Therefore, the SCT layer is directly exposed to chronic elevated glucose levels. In human trophoblast primary culture from normal placentas, incubation with glucose at concentrations comparable to in vivo hyperglycemic concentration provokes increased MDA production [99]. In cultured mouse conceptuses, incubation in high glucose conditions led to a marked increase of intracellular ROS [100].

Hyperglycemia-mediated oxidative stress causes distinct perturbations in placental physiology. Firstly, it influences glucose uptake from maternal circulation. GLUT1 is a major glucose transporter in the placenta and in normal conditions; it is most highly expressed on the membrane of the SCT, but loses this localization in the case of GDM [101]. When primary trophoblasts from normal placentas are incubated in hyperglycemic-like conditions, a GLUT1 mRNA decrease is observed, positively correlated with oxidative stress [99]. Additionally, in the BeWo cell line, oxidative stress induced by exposure to a pro-oxidant molecule led to reduced [^3^H]-deoxyglucose uptake, possibly by modulating GLUT1 post-transcriptionally [102]. Oxidative stress in GDM placentas also has an effect on the trophoblast potential; in mouse blastocysts cultured in hyperglycemic conditions, rising ROS appears to regulate trophoblast spreading. An increase of trophoblast outgrowth was observed, as well as an increase in MMP9 expression and plasminogen activity [100].

It was also proposed that GDM placentas undergo adaptation to chronic oxidative stress. Explants of human postpartum placentas were exposed to acute oxidative stress by the presence of hypoxanthine/xanthine oxidase in the cell culture medium, and GDM failed to increase the expression of antioxidant enzymes and inflammatory cytokines [103]. This result suggests that the placenta does play the role of buffer between maternal and fetal circulation when GDM is present, in which case it is more strongly solicited and renders supplementary adaptation to a new stress difficult.

## 4. Role of Oxidative Stress on the Maternal Endothelial Dysfunction in Preeclampsia

### 4.1. Preeclampsia: An Oxidative Stress-Mediated Inflammatory Disease of the Maternal Endothelium

Vascular endothelium consists of a single layer of epithelial cells covering the interior surface of blood vessels. This acts as a semi-selective barrier between the vessel lumen and the more exterior tunics of the vessel wall (media and externa), thus controlling the passage of molecules in and out of the bloodstream. Additionally, the vascular ECs also have paracrine and autocrine functions, allowing them to modulate arterial vasomotility (vasoconstriction/dilatation), leukocyte adhesion and diapedesis, and platelet coagulation and fibrinolysis, as well as proliferation and differentiation of smooth muscle cells in the tunica media [104]. The direct contact of vascular ECs with the blood flow makes them ideal sensors of circulating factors, yet at the same time, makes them particularly sensitive to circulating damaging factors. Thus, endothelial cell dysfunction is a pivotal event in the development of vascular diseases.

Numerous studies have brought evidence to support the theory that maternal PE is a consequence of vascular endothelial dysfunction induced, at least in part, by factors released from an ischemic placenta [105]. In PE, both glomerular endotheliosis of the kidney and structural modifications of the umbilical vein and placental uterine vessel endothelium have been observed. Furthermore, in preeclamptic women, a decrease in endothelium-dependent vasodilation has been demonstrated using non-invasive techniques. Additionally, preeclamptic plasma contains increased levels of various markers of endothelial activation, including adhesion molecules, cytokines, and pro-coagulant and anti-angiogenic factors (sFLT-1 and sENG). Therefore, most maternal symptoms of PE can be explained as a result of endothelial dysfunction. For example, endothelial glomerulosis is most likely the etiology of proteinuria. Deficient, endothelium-dependent vasodilation causes hypertension and triggers vasoconstriction in different organs, causing hypoperfusion and ischemia. Activation of ECs also leads to systemic inflammation and edema. The activation of ECs by inflammatory substances promotes leukocyte adhesion and permeability disorders. EC’s surface expresses leukocyte adhesion molecules such as intercellular adhesion molecule-1 (ICAM-1), vascular cell adhesion molecule-1 (VCAM-1), E-selectin (SELE), and P-selectin (SELP). Leukocytes then adhere to ECs via these surface molecules and produce ROS and proteases, which cause endothelial damage. A number of experimental data suggest that ROS play an important role in regulating the production of leukocyte adhesion molecules in ECs [10]. The expression of ICAM-1, VCAM-1, and the chemoattractant protein-1 (MCP-1) is induced by TNF-α and controlled by an ROS-dependent mechanism [106]. In fact, this induction can be inhibited by the use of antioxidants and the inhibition of the NOX [107]. The expression of SELP is also induced by TNF-α, and is associated with the production of ROS by both NOX and XO [108]. EC damage causes them to lose the structural properties of their membranes, and results in cellular edema, as well as plasma leakage from the vessel lumen to the interstitial space. The effect of TNF-α and thrombin has been clearly demonstrated as the causal agent of increased endothelial permeability [109]. Vascular edema causes decreased capillary hydrostatic pressure and increased colloidal osmotic pressure (due to increased blood viscosity), which alters capillary perfusion and leads to hypoxia [110]. The formation of endothelial edema is directly related to intracellular calcium concentrations. ROS disrupts the activity of enzymes controlling the intracellular influx of calcium, such as phospholipase C (PLC), as well as the enzymatic activity governing intracellular calcium fluxes, such as the calcium-ATPase of the endoplasmic reticulum (SERCA) [111]. The resulting interstitial edema increases ischemia, leading to further production of ROS and thus creating a self-perpetuating cycle.

**Figure 4:**
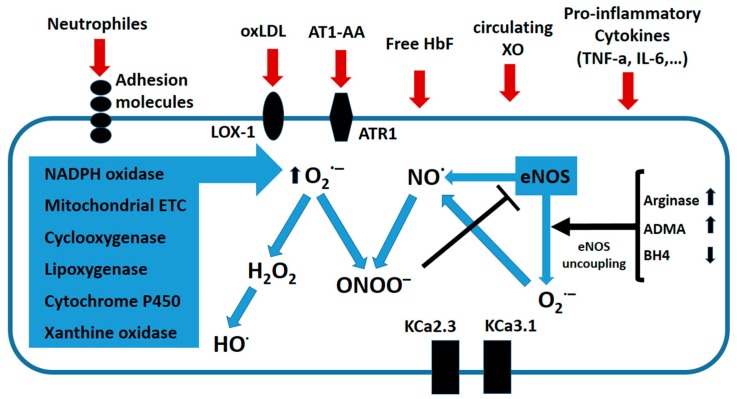
Principal mediators and sources of oxidative stress in the preeclamptic endothelium. Circulating factors in the blood of preeclamptic women can act on endothelial cells (ECs) to induce oxidative stress. These include reactive oxygen species (ROS) produced by neutrophils (oxLDL), agonist autoantibodies against angiotensin receptors (AT1-AA), free fetal hemoglobin (HbF), circulating Xanthine oxidase (XO), and cytokines (i.e., TNF-α). In ECs, several enzymatic systems including the electron transport chain, NADPH oxidases, and cyclooxigenases can produce superoxide (O_2_^•−^). Under certain circumstances, this can lead to increased Arginase II expression, increased asymmetric dimethyl arginine (ADMA), and the loss of the cofactor tetrahydrobiopterin (BH4), and endothelial nitric oxide synthase (eNOS) synthase can become uncoupled. Instead of •NO, uncoupled eNOS produces (O_2_^•−^). Nitric oxide can then react with O_2_^•−^ to produce peroxynitrite (ONOO^−^), a powerful oxidant whose nitrate proteins can induce DNA damage. Additionally, ONOO^−^ can inhibit eNOS activity. Superoxide scavenging of •NO impairs endothelium-dependent vasodilation. ROS can also down regulate the calcium-activated potassium channels KCa2.3 and KCa3.1, which are important electrical triggers of vasodilation. Figure adapted from [105].

Therefore, most symptoms of PE result from a systemic dysfunction of the maternal endothelium caused by anti-angiogenic molecules released by the placenta [5,112]. Several components in the plasma of preeclamptic women (cytokines, activated neutrophils, peroxidized lipids, xanthine oxidase, fetal hemoglobin, etc.) can act on ECs and trigger oxidative stress (Figure 4).

As previously seen, cytokines, such as TNF-α, are present at higher levels in preeclamptic plasma, and can initiate OS in ECs. TNF-α directly induces oxidative damage through the activation of NOX, which then leads to the production of superoxide anions that can scavenge •NO [113]. It can also stimulate mitochondrial OS; TNF-α activates free radical production at the ubiquinone site and damages the mitochondrial ETC at complex III, resulting in increased production of ROS [114]. Furthermore, TNF-α and other circulating factors present in PE plasma can induce oxidative stress in ECs indirectly by up-regulating the Lectin-like oxidized LDL receptor-1 (LOX-1) and a receptor for oxidized LDL (oxLDL) [115]. In ECs, up-regulation of LOX-1 results in increased oxLDL uptake, which then leads to increased production of O_2_^•−^ via activation of NOX [116]. TNF-α has been suspected to also increase production of free radicals through the activation of XO, although this seems to be controversial [106]. The NOX are a major source of O_2_^•−^ generation in ECs, and many circulating factors that are increased in the context of PE are capable of activating NOX. In vitro studies have shown that HUVEC cells treated with serum from preeclamptic women overexpress the subunit NOX2, which results in high amounts of O_2_^•−^ [117]. In addition, overexpression of NOX2 was seen in primary cultures of HUVECs isolated from preeclamptic pregnancies when compared to those cells from non-pathological pregnancies [118]. Additionally, agonist autoimmune antibodies against the angiotensin II receptor type I (AT1-AA) have been detected in the blood of preeclamptic women [119]. Since then, circulating AT1-AAs have been found in patients with many other cardiovascular diseases. Moreover, several experimental studies have demonstrated that AT1-AA participates in the pathogenesis of PE [120]. Stimulation of AT1 receptor by AT1-AA in vitro results in the inhibition of trophoblast invasiveness. Also, AT1-AA can activate the AT1 receptor in ECs, vascular smooth muscle cells, and renal mesangial cells. Administration of AT1-AA to pregnant rats induces a full spectrum of PE symptoms including hypertension, proteinuria, glomerular capillary endotheliosis, and increased production of sFLT1 and sENG. These effects of AT1-AA are the direct consequence of AT1R activation. This promotes a series of cellular responses, including the release of ET-1 and the activation of NOX. Then, NOX activation leads to increased production of ROS, which can, among other effects, impair endothelial-dependent dilation by scavenging •NO.

In ECs, another effect of the NOX-mediated increase in OS is down regulation of the calcium-activated potassium channels, KCa2.3 and KCa3.1 (KcaS). These channels are important electrical triggers in vasomotor activity that contribute significantly to endothelium-dependent relaxation (EDR). Thus, up-regulation of KcaS promotes EDR, whereas its down-regulation enhances vascular contractility and elevates blood pressure. Oxidative stress indeed modulates the expression of these channels. A recent in vitro study has shown that treatment of ECs with PE serum, ox-LDL, progesterone, or sFlt-1 all leads to depressed KCas levels. This inhibition seems to be the consequence of increased O_2_^•−^ production via high NOX2 and NOX4 expression and reduced SOD levels [121,122].

The •NO radical is in fact a powerful vasodilator that induces relaxation of smooth muscles. However, eNOS can also generate O_2_^•−^ and H_2_O_2_, particularly if the intracellular concentrations of l-arginine or its cofactor, BH4, are low [123,124]. HUVECs treated with 2% preeclamptic plasma show increased expression and activity of arginase II, which, in turn, reduces the availability of l-arginine. PE plasma is also known to increase production of O_2_^•−^ and ONOO^−^ in the same cells. However, inhibition of either arginase II or eNOS decreases O_2_^•−^ production. Thus, arginase II can lead to the uncoupling of eNOS, reducing •NO production and favoring O_2_^•−^ production [125]. In biological systems, ONOO^−^ reacts primarily with tyrosine residues in proteins to produce 3-nitrotyrosines [126]. Nitration of proteins can alter both their structure and function. In addition, ONOO^−^ can also cause DNA damage and lipid peroxidation. In ECs, ONOO^−^ increases iNOS expression through an NF-kB-dependent mechanism and inhibits prostacyclin synthase [127]. Furthermore, ONOO^−^ also promotes production of the vasocostrictor ET-1 [112]. In the placenta, residues of 3-nitrotyrosine have been observed in both normal and preeclamptic pregnancies, mainly in the endothelium that surrounds smooth muscle and villous stroma [65].

Many factors present in preeclamptic circulation can activate LOX-1, including anionic phospholipids, platelets, cytokines, and debris from apoptotic cells. Thus, LOX-1 is highly expressed in the arteries of preeclamptic women and oxidized LDL is found to be elevated in their plasma. When HUVECs were treated for 24 h with 2% plasma from preeclamptic women, an increase of LOX-1 expression and oxidized LDL uptake was observed. This resulted in OS overproduction, as evidenced by increased NOX activity, O_2_^•−^, and ONOO^−^. Peroxynitrite specifically is able to up-regulate LOX-1 expression, suggesting the presence of a feedback mechanism in which LOX-1 activation induces oxidative stress, which in turn induces LOX-1 [128]. However, other mechanisms have been proposed to explain LOX1 induction in preeclamptic ECs. One such mechanism involves Methylglyoxal (MG), a highly reactive molecule that reacts with various amino acid residues in proteins to produce advanced glycation end products (AGEs). MG has, in fact, been detected in the vasculature of women with PE. Furthermore, semicarbazide-sensitive monoamine oxidase (SSAO)-, the enzyme that generates MG, is increased in PE cases, whereas the expression of glyoxalase enzymes that normally degrades MG is reduced. In cultured ECs, MG progressively increased arginase II and LOX-1 expression. When either arginase II or NOS was inhibited, significant reductions in MG-induced LOX-1 expression and O_2_^•−^ levels, and nitrotyrosine staining was observed. Thus, MG can induce LOX-1 expression via arginase II up-regulation, likely through NOS uncoupling [129]. Another mechanism proposed for LOX-1 induction has been described in mouse aortas, and involves Toll Like Receptor 4 (TLR4) signaling via a p38 mitogen-activated protein kinase (MAPK/NF-kB) pathway [130].

Factors released by the preeclamptic placenta can also affect indirectly the ECs by acting on circulating cells. Thus, in PE, the production and release of O_2_^•−^ and H_2_O_2_ from circulating neutrophils increases and leads to EC injury. In vitro studies using HUVECs indicate that this neutrophil-mediated damage model involves reduced bioavailability of •NO and ONOO^−^. [131]. In addition, production of O_2_^•−^ and its derivative, H_2_O_2_, by neutrophils results in increased neutrophilic CD11b expression and adhesion to ECs [132].

### 4.2. Antioxidant Therapeutic Approaches to Treat Endothelial Dysfunction in PE

The importance of OS in PE-induced endothelial dysfunction has directed the development of therapeutic approaches aiming to restore the redox equilibrium. Many studies have focused on antioxidant supplementation as a treatment for PE. These act as protective agents to prevent oxidative damage caused by ROS [103]. However, comparative studies seeking to evaluate circulating levels of these agents in preeclamptic and normal plasma show contradictory results [133,134,135]. Some studies have reported decreased SOD and catalase activity in PE women, whereas others report either increased or decreased SOD and higher catalase activities. Similarly, some studies have reported lower GSH in preeclamptic plasma, while others reported unchanged levels. Also, the •NO levels in the plasma of women with PE remain controversial [134]. Several studies suggest that •NO levels are similar to non-pathological pregnancies, while other studies have found either higher or lower levels. In preeclamptic women, plasma concentrations of l-arginine (the substrate of eNOS) have been found significantly decreased, whereas ADMA levels (an eNOS inhibitor) have been found to be unchanged or even increased in some studies [134]. Either reduced l-arginine and/or increased ADMA have the potential to limit the bioavailability of •NO in the maternal vascular system. Supplementation with antioxidants such as vitamins C and E has been explored as an avenue to prevent EC dysfunction in PE. Vitamin C scavenges free radicals and Vitamin E prevents lipid peroxidation. However, exhaustive review of the studies seeking to evaluate their utility has concluded that the experimental evidence does not support the use of these vitamins for PE treatment. [136]. By contrast, epidemiological and clinical studies, followed by systematic reviews, have consistently shown an inverse relationship between high blood pressure and calcium. These studies suggested that a calcium supplement could lower the risk of PE [137]. Therefore, the World Health Organization (WHO) has recommended for pregnant women to supplement calcium, especially those in high-risk populations, with a low calcium diet [138]. Vitamin D insufficiency has also been associated with PE [139]. Since vitamin D deficiency is exacerbated by lack of sunlight exposure, it has been correlated with higher PE incidence in northern countries with scarce sunlight hours/day in the winter months. Vitamin D also plays a crucial role in calcium homeostasis, and there are several hypotheses proposing how vitamin D deficiency can increase the risk of PE. These include its role in modulating pro-inflammatory responses and decreasing OS, promoting angiogenesis through VEGF and gene modulation, and decreasing blood pressure through the renin-angiotensin system (RAS). Despite the fact that vitamin D insufficiency in pregnancy has been linked to adverse maternal and fetal outcomes, its role and therapeutic utility for PE prevention remain controversial [140]. Nevertheless, vitamin D is reported to induce angiogenesis in endothelial progenitor cells and to play a major role in the EC function or dysfunction in cell culture models [141]. Additionally, the release of OS-induced endothelial microparticles (MPs) is found to be elevated in numerous cardiovascular diseases, including PE. These MPs have pro-coagulant and pro-inflammatory properties and are good indicators of EC dysfunction. A recent in vitro study has shown that Vitamin D suppresses endothelial MP release through a mechanism involving OS modulation [142]. Moreover, vitamin D may alleviate oxidative stress by competitive inhibition of placental cytochrome P450ssc, which prevents the production of lipid peroxides and/or excess progesterone synthesis, both of which may contribute to the physiopathology of PE [2]. Clinical studies also suggest that high-dose supplementation with vitamin D (4000 IU/day) during pregnancy can practically remove the risk of PE.

In addition to the use of supplemental antioxidants, other therapeutic approaches aim to either activate EC’s antioxidant mechanisms and/or to mitigate the effects of ROS production. Thus, a recent study has shown that Sofalcone (a gastric antiulcer agent) can also suppress endothelial dysfunction [143]. In primary HUVECs, solfacone treatment increased HO-1 expression. Furthermore, it caused translocation of the nuclear factor (erythroid-derived 2)-like 2 (NFE2L2 also known as NRF2) and transactivation of other NFE2L2-responsive genes (NQO1, TXN, and GCLC). In these cells, Solfacone also blocks TNF-α-induced monocyte adhesion and VCAM-1 expression. These results indicate that Sofalcone can activate an antioxidant nuclear factor NFE2L2/HO-1 pathway and thus ameliorate endothelial dysfunction. Preclinical studies using animal models have shown that oral Sofalcone administration has no adverse effects on pregnancy. Consequently, this is a promising novel therapeutic candidate for PE. Another study by the same team has also explored the use of Proton Pump Inhibitors (PPIs) to treat endothelial dysfunction in PE [144]. PPIs decrease sFlt-1 and soluble endoglin (sENG) secretion from trophoblasts, placental explants from preeclamptic pregnancies, and ECs. They also mitigate TNF-α-induced endothelial dysfunction, PPIs blocked VCAM-1 expression, leukocyte adhesion to endothelium, and disruption of endothelial tube formation. They also decrease ET-1 secretion and enhanced ECs migration. Interestingly, the PPI Esomeprazole can dilate maternal blood vessels of normal pregnancies, as well as in cases of severe PE. Moreover, Esomeprazole was able to decrease blood pressure in a transgenic mouse model in which human sFlt-1 was overexpressed in the placenta. In addition, PPIs up-regulated endogenous antioxidant defenses and decreased cytokine secretion in placental tissue and ECs. Moreover, proton pump inhibitors (PPIs) are safe for use in pregnancy, in which they are often prescribed for gastric reflux.

The oxidative stress generated by the mitochondria is a new potential target with which to address the vascular dysfunction of PE. HUVECs exposed to 3% plasma from women with PE pregnancies resulted in a significant decrease in mitochondrial function with subsequent significant increases in mitochondrial O_2_^•−^ production when compared to cells exposed to plasma from women with non-pathological pregnancies. Also, increased expression of inflammatory markers TNF-α, TLR-9, and ICAM-1 was detected in ECs treated with PE plasma. Pre-treatment of these ECs with MitoTempo (a mitochondria-targeted superoxide dismutase antioxidant mimetic) provided protection against H_2_O_2_-induced cell death. Furthermore, MitoTempo significantly reduced mitochondrial O_2_^•−^ production in cells exposed to PE plasma by normalizing mitochondrial metabolism. It also modified the inflammatory profile of PE plasma-treated ECs. These findings support the role of mitochondrial redox signaling by modulating EC dysfunction in the context of PE, and point to mitochondrial-targeted antioxidants as potential therapeutic candidates [145].

In PE, free fetal hemoglobin (HbF) leaks to the maternal circulation, where it causes endothelial damage and vasoconstriction [146]. This vasoconstrictive effect of free HbF is the result of strong binding of ferrous HbF to •NO, thereby reducing its availability. In addition, O_2_–bound ferrous HbF generates ROS. Furthermore, its degradation products, heme and free iron, are toxic and induce an inflammatory response by activating neutrophils and cytokine production. Ex vivo and in vivo studies have shown that the protein Alpha 1-microglobulin (A1M), with free radical and heme binding properties, has the capacity to counteract free Hb-induced placental and glomerular endotheliosis [147,148]. Thus, A1M could be used as a scavenger to mitigate the effects of free HbF and ROS in PE.

Resveratrol is known to modulate pathways that involve inflammation and OS, and thus could be a potential therapeutic avenue for PE. It reduces sFlt-1 and sENG secretions from both primary trophoblasts and HUVECs, and also decreases mRNA expression of pro-inflammatory molecules [149]. In HUVECs, resveratrol significantly increased the expression level of anti-oxidant enzymes HO-1, NQO1, GCLC, and TRX, but failed to significantly alter HO-1 expression. It did, however, reduce HO-1 proteins in trophoblast. Furthermore, resveratrol enhances the effect of TNFα, which increases HUVEC’s expression of VCAM1 and adhesion of peripheral blood mononuclear cells. In contrast, resveratrol significantly reduced TNFα-induced ET-1, yet to the same degree increased eNOS phosphorylation. Thus, resveratrol is able to decrease secretion of anti-angiogenic factors; however, its effects on ECs are mixed. Therefore, its potential as a treatment for PE appears to be uncertain at this time.

The Renin-Angiotensin System has been intensively analyzed in the context of PE and envisaged as a potential therapeutic target. Normal pregnant women show relative insensitivity to direct infusion of the vasopressor hormone angiotensin II (AngII), which is considered a physiological adaptation that promotes low systemic vascular resistance. On the contrary, preeclamptic women display increased sensitivity to AngII [150]. This could be explained by the decrease in levels of vasodilatatory angiotensin 1–7 (Ang 1–7), or by the presence of AT1R-activating autoantibodies (AT1-AA). Among other effects, signaling trough the ATR1 receptor leads to increased production of ROS and subsequent reduced NO bioavailability. It has been shown that the administration of agonistic AT1-AAs from preeclamptic women to mice induces the hallmark symptoms of PE (hypertension, proteinuria, etc.), which can all be attenuated by the AT1R antagonist, Losartan. Unfortunately, Losartan, as with other AT1R blockers and angiotensin-converting enzyme inhibitors (ACEIs), which are commonly used to treat hypertension, cannot be used to treat PE due to their documented toxicity during pregnancy [150].

Recently, Burke et al. have established a link between sFLT1 and increased sensitivity to Ang II in PE [151]. Using sFlt1-overexpressing pregnant mice, they show that an excess of sFLT1 is sufficient to induce vascular sensitivity to AngII. Moreover, sFLT1 acts on the maternal endothelium to inhibit eNOS phosphorylation at Ser 1177 (which is required for enzymatic activity; see Section 2.1). This is associated with increased blood pressure, vascular oxidative stress, and sensitivity to vasopressors. Sildenafil, a phosphodiesterase 5 (PDE5) inhibitor that raises cGMP levels, is sufficient to restore AngII resistance during pregnancy and inhibit sFLT1-mediated hypertension. Treatment with Sildenafil restores eNOS activity, reduces OS, and attenuates vasoconstriction. In addition, Sildenafil treatment improves uterine blood flow, decreases uterine vascular resistance, and improves fetal weight. In their study, the authors report that Sildenafil treatment appears to be safe, since it does not induce toxic effects in the mothers or fetuses. Thus, Sildenafil could potentially be very effective in the treatment of endothelial dysfunction in PE.

Aspirin (acetylsalicylic acid) holds also the potential to treat OS and NS in the context of PE. It has been reported that before the 16th week of pregnancy, daily administration of aspirin at low doses (150 mg/day) reduces the risk of PE [152]. The putative mechanism of action of aspirin in the context pregnancy complications has been recently reviewed [153]. Aspirin is a non-steroidal anti-inflammatory drug (NSAID). Its pharmacological properties are due to its reactive salicylate and acetate groups, both being biologically active and acting independently of each other at different sites [154,155]. Aspirin exerts many pharmacological actions including the irreversible inhibition of COX1 and the modification (by acetylation) of the enzymatic activity of COX2, leading to the suppression of prostaglandins and thromboxanes. Aspirin prevents also the activation of genes of the inflammatory pathway response by blocking the activation of NF-κB. These anti-platelet and anti-inflammatory actions have beneficial effects for the prevention of atherosclerosis, heart disease, stroke, and some cancers. In addition, a number of studies suggest that aspirin can reduce OS and NS. Aspirin acetylation of eNOS elicits NO release by the vascular endothelium [156]. Also, in ECs, aspirin induces enzymatic activity of the HO-1 and thus contributes to reducing OS [157]. Another mechanism involves the production of aspirin-triggered lipoxins (ATLs) from arachidonic acid by acetylation of COX2. ATLs produced by COX2-expressing cells (including monocytes and macrophages) act as mediators, promoting the resolution of inflammation, and act also as antioxidant and immunomodulators. Moreover, ATLs block the generation of ROS species in ECs by a mechanism that could induce NO synthesis through both iNOS and eNOS [158,159]. Another study has shown that in rat brain tissue subjected to hypoxia, oral administration of aspirin reduces OS through a mechanism involving increased GSH level and GPx activities and reduced iNOs activity [160]. However, salicylic acid produced greater reduction in OS and iNOs activities than aspirin, which may indicate that the salicylate moiety of aspirin may have a stimulatory effect on GSH biosynthesis and subsequently modulates related antioxidant enzymes activities. Thus, there is substantial experimental evidence suggesting that aspirin, either directly or not, has the potential to contribute to modulate OS and NS in PE. Further research is needed to clarify the mechanisms of action of aspirin in the context of PE and PE-induced OS and NS to better rationalize the use of this molecule.

## 5. Conclusions

Normally, oxygen is a vital gas that enables our cells to function via the production of mitochondrial ATP. Despite its essential physiological role, however, molecular oxygen is a potentially toxic biradical. In extreme cases, breathing high concentrations of oxygen lead to hyperoxia, which causes CNS, pulmonary, renal, hepatic, and ocular toxicity due to the disruption of cell membranes and other cell molecules. The impacts on the cells are assumed to be associated with oxidative stress and are mediated by ROS.

In placental mammals, the placenta is a transient organ, a very important function of which is to manage the exchanges of nutrients, oxygen, and waste between the mother and fetus. An additional level of complexity is given by the fact that in humans, a hypoxic state must be maintained in the first trimester, while later the oxygen pressure rises to that of ‘normal’ tissue conditions. This implies that a very tight regulation of oxygen pressure has to be maintained in the placenta, and this process, which involves thousands of genes and numerous steps, allows for multiple opportunities for error. These dysfunctions become either causes or consequences of placental-based diseases, such as IUGR, spontaneous pre-term birth, GDM, and, most importantly, PE. In a recent perspective, Seki presented PE symptoms as a series of dominos in varying hierarchical positions [161]. In the cascade of events leading to eclampsia, HELLP, Premature Birth, or other consequences for the mother and child, placental OS is placed in a very high position, just below placental ischemia due to defective spiral artery remodeling caused by immune maladaptation. In the present study, we attempted to elucidate a more complete vision of this syndrome, showing the origin of oxidative stress in the placenta and endothelial cells, as well as the details of its consequences. A deeper understanding of the mechanisms governing oxidative stress in both the placenta and maternal vascular endothelium is likely to offer new opportunities for the development of innovative therapeutic approaches to treat preeclampsia, thus saving over 70,000 maternal lives per year and improving the later life quality of newborns.

## Abbreviations

A1M	Alpha 1-microglobulin
ADMA	asymmetric dimethyl arginine
AEGs	advanced end glycation products
AngII	angiotensin II
AOPPS	advanced oxidation proteins products
AT1-AA	Agonist autoimmune antibodies against the angiotensin II receptor type I
ATLs	Aspirin-triggered lipoxins
BH4	tetrahydrobiopterin
CAT	catalase
CBS	cystathionine-β-synthase
COX1	Cyclooxygenase 1 = PTGS1 (Prostaglandin synthase 1)
COX2	Cyclooxygenase 2 = PTGS2 (Prostaglandin synthase 2)
CSE	cystathionine-γ-lyase
ECs	endothelial cells
EDR	endothelium-dependent relaxation
ENG	Endoglin
eNOS	endothelial nitric oxide synthase
ETC	electron-transport chain
EVT	extravillous trophoblasts
EVT	extravillous trophoblasts
GDM	Gestational diabetes mellitus
GPx	glutathione peroxidase
GSH	Glutathione
H_2_O_2_	hydrogen peroxide hydroxyl radical
HbF	free fetal hemoglobin
HCG	Chorionic Gonadotropin
*HMOX1*	Heme Oxygenase 1
HUVEC	Human Umbilical Vascular Endothelial Cell
HO-1	hemoxygenase
•HO	hydroxyl radical
ICAM-1	intercellular adhesion molecule-1
iNOs	inducible nitric oxide synthase
IUGR	Intra-uterine Growth Restriction
KcaS	calcium-activated potassium channels
LOX-1	LDL receptor-1
MDA	Malondialdehyde
MG	Methylglyoxal
MMPs	Matrix metalloproteinases
NADH	Nicotinamide adenine dinucleotide
NF-κB	nuclear factor-kappa B
nNOS	neuronal nitric oxide synthase
•NO	nitric oxide
NOX	NADPH oxidase
NSAID	Non-Steroidal Anti-Inflammatory Drug
O_2_^•−^	superoxide
ONOO^−^	peroxynitrite
OS	Oxidative Stress
oxLDL	oxidized LDL
PC2	polycystin-2
*PDCD1LG2*	Programmed Cell Death 1 Ligand 2
*PDGFD*	Platelet Derived Growth Factor D
PIF	Preimplantation factor
PLC	phospholipase C
PPIs	Proton Pump Inhibitors
PRKG1B	Protein Kinase, CGMP-Dependent, Type I
RAS	renin-angiotensin system
RNS	reactive nitrogen species
ROS	reactive oxygen species
ROS	reactive oxygen species
SCT	syncytiotrophoblasts
SELE	E-selectin
SELP	P-selectin
sENG	soluble endoglin
SERCA	calcium-ATPase of the endoplasmic reticulum
sFlt1	soluble fms-like tyrosine kinase-1
SOD	superoxide dismutase
SSAO	semicarbazide-sensitive monoamine oxidase
TBARS	Thiobarbituric acid reactive substances
*TGFBR2*	Transforming Growth Factor Beta Receptor 2
TRX	Thioredoxin
VCAM-1	vascular cell adhesion molecule-1
VCT	villous cytrophoblasts
*VLDLR*	Very Low Density Lipoprotein Receptor
XO	Xanthine Oxydase
